# The Role of Vitamin D as a Biomarker in Alzheimer’s Disease

**DOI:** 10.3390/brainsci11030334

**Published:** 2021-03-06

**Authors:** Giulia Bivona, Bruna Lo Sasso, Caterina Maria Gambino, Rosaria Vincenza Giglio, Concetta Scazzone, Luisa Agnello, Marcello Ciaccio

**Affiliations:** 1 Institute of Clinical Biochemistry, Clinical Molecular Medicine and Laboratory Medicine, Department of Biomedicine, Neurosciences and Advanced Diagnostics, University of Palermo, 90127 Palermo, Italy; giulia.bivona@unipa.it (G.B.); bruna.losasso@unipa.it (B.L.S.); cmgambino@libero.it (C.M.G.); rosaria.vincenza.giglio@alice.it (R.V.G.); concetta.scazzone@unipa.it (C.S.); luisa.agnello@unipa.it (L.A.); 2 Department of Laboratory Medicine, AOUP “P. Giaccone”, 90127 Palermo, Italy

**Keywords:** Alzheimer’s Disease, Vitamin D, 25(OH)D levels, biomarker, Vitamin D deficiency

## Abstract

Vitamin D and cognition is a popular association, which led to a remarkable body of literature data in the past 50 years. The brain can synthesize, catabolize, and receive Vitamin D, which has been proved to regulate many cellular processes in neurons and microglia. Vitamin D helps synaptic plasticity and neurotransmission in dopaminergic neural circuits and exerts anti-inflammatory and neuroprotective activities within the brain by reducing the synthesis of pro-inflammatory cytokines and the oxidative stress load. Further, Vitamin D action in the brain has been related to the clearance of amyloid plaques, which represent a feature of Alzheimer Disease (AD), by the immune cell. Based on these considerations, many studies have investigated the role of circulating Vitamin D levels in patients affected by a cognitive decline to assess Vitamin D’s eventual role as a biomarker or a risk factor in AD. An association between low Vitamin D levels and the onset and progression of AD has been reported, and some interventional studies to evaluate the role of Vitamin D in preventing AD onset have been performed. However, many pitfalls affected the studies available, including substantial discrepancies in the methods used and the lack of standardized data. Despite many studies, it remains unclear whether Vitamin D can have a role in cognitive decline and AD. This narrative review aims to answer two key questions: whether Vitamin D can be used as a reliable tool for diagnosing, predicting prognosis and response to treatment in AD patients, and whether it is a modifiable risk factor for preventing AD onset.

## 1. Introduction

If one searches for the keywords “Vitamin D” and “Cognition” in Pubmed.com, one finds over 1000 articles that have been published with no break in continuity for the past 50 years. The idea of a possible link between Vitamin D metabolism and brain function has been successfully proposed and then proved by a remarkable body of data. When assessing the Vitamin D circulating levels in Mild Cognitive Impairment and Alzheimer Disease (AD) patients, an association has yet been found. Nevertheless, the attempt to use Vitamin D as a biomarker of cognitive decline systematically failed and, furthermore, Vitamin D supplementation in these patients yielded controversial results. Many reasons can explain this debacle. First, the studies assessing Vitamin D levels and its serum biomarker 25(OH)D in AD patients have some limitations (different assay methods; heterogeneity of Vitamin D cut-offs; discrepancies among the measures used to define the cognitive function), which sharply limit the robustness of findings achieved. Second, discrepancies in the cut-offs and methods used to measure 25(OH)D across the studies, due to the lack of 25(OH)D measurement standardization, made the results difficult to interpret. Third, a specific biomarker’s clinical usefulness is defined as its capability to influence clinicians to diagnose the disease, predict prognosis, and guide treatment, which is nothing Vitamin D can do. Indeed, well-established diagnostic biomarkers for AD are currently available. Hence, there is no need for a marker for diagnosis, and, on the other hand, effective treatment for AD lacks so far. Therefore, it is unclear whether Vitamin D circulating levels can impact AD patients’ outcome until a question is addressed: is AD onset preventable by reaching the optimal Vitamin D levels? Based on the available literature data, this review aims to explain why this question’s answer could be no.

Vitamin D is a steroid hormone that can be synthesized endogenously. Primarily known to regulate calcium-phosphorus metabolism, it exerts several biological activities, counting brain function and immune response regulation [1,2,3].

In humans, Vitamin D is produced in a multi-step process that involves the ultraviolet B (UVB) rays irradiation of a cutaneous compound, the 7-dehydro-cholesterol (7-DHC). 

Once UVB rays act on 7-DHC, the cholecalciferol is produced, needing two sequential hydroxylation steps to form the active Vitamin D. First hydroxylation occurs in the liver, by a 25 hydroxylase generating 25(OH)D, while the second mainly depends on a renal 1,25 hydroxylase, producing 1,25(OH)_2_D. 1,25 hydroxylase is present within various organs and cells; thus, Vitamin D’s active form can be produced in several tissues, including the lung, brain, prostate, placenta, and immune system cells. CYP2R1, CYP3A4, and CYP27A1 enzymes have 25-hydroxylase activities, while CYP27B1 is responsible for 1,25 hydroxylation. Kidney CYP27B1 gives rise to a hormone involved in calcium-phosphorus metabolism. Non-renal active Vitamin D is implicated in regulating some cellular processes, including cell differentiation and proliferation. While CYP27B1 is regulated by the parathyroid hormone (PTH), the fibroblast growth factor (FGF23) and 1,25(OH)_2_D, extra-renal CYP27B1 is regulated by interferon γ (IFN-γ) and tumour necrosis factor (TNF) [4,5]. 

Vitamin D binding protein (VDBP) conveys both 25(OH)D and 1,25(OH)_2_D from the liver and kidney to other tissues, where active Vitamin D binds the nuclear Vitamin D Receptor (VDR) [3,6,7,8,9], leading to the genomic and non-genomic actions (for more details on Vitamin D genomic and non-genomic actions see reference 1). 

CYP24A1 enzyme, displaying 24 hydroxylase activity, carries out Vitamin D catabolism.

Vitamin D status is typically evaluated by measuring serum 25(OH)D [9]. A consensus on which 25(OH)D levels define Vitamin D sufficiency, deficiency, and insufficiency is lacking, also due to the standardization dearth in the past decades [10]. Most of the studies performed on Vitamin D’s role in various diseases report unstandardized data, and AD is no exception. Thus, Vitamin D’s reliability as a serum biomarker in AD has been considered a debatable issue, leading to controversial opinions across the scientific community [10]. 

## 2. Vitamin D and Alzheimer Disease

A growing interest in Vitamin D role in both brain development and function in adulthood led several authors to investigate the 25(OH)D circulating levels in AD patients [11,12,13,14,15,16,17,18,19,20,21,22,23,24,25,26]. The brain displays the capability to produce and receive Vitamin D’s active form, which is deemed to support neurotransmission, synaptic plasticity, and neuroprotection [1,2,10]. From a pathophysiologic point of view, the relation between Vitamin D and AD onset and progression has been explained by impressive in vitro and in vivo studies. Given that amyloid plaques, along with neurofibrillary tangles, represent features of AD, it has been shown that 1,25(OH)_2_D can help the amyloid plaques phagocytosis and clearance by the innate immune cells [1,2,27,28,29,30,31]. For instance, MCI and AD patient-derived macrophages show enhanced capability to eliminate amyloid plaques after 1,25(OH)_2_D treatment [30], and a Vitamin D-enriched diet can decrease the number of plaques in AβPP-PS1 transgenic mice, an AD animal model [31]. Also, amyloid protein precursor (APP) metabolism involves some transcription factors, counting SMAD and transforming growth factor-beta (TGF-β), that, in turn, interact with VDR/ligand complex in the nucleus [29,32,33]. Finally, it should be considered that Vitamin D has a role in reducing cerebral microenvironment inflammation and oxidative stress, which are regarded as possible mechanisms underlying neurodegeneration and AD pathogenesis [1,10,29]. Table 1 summarises the characteristic of the studies considered.

### 2.1. Observational Studies on 25(OH)D Serum Levels in AD Patients

Based on these considerations, Littlejohns et al. [14] enrolled, in 2014, 1658 subjects, of which 171 developed dementia (102 AD out of 171 all-cause dementia) over a 5.6-year follow-up period. Findings revealed that subjects with 25(OH)D serum levels <25 nm/L had a two-fold risk of AD onset compared to those with >50 nm/L. Authors defined Vitamin D deficiency <50 nm/L, distinguishing between deficiency and severe deficiency (25 to 50 nmol/L and <25 nm/L, respectively). The strength of the study was the use of procedures and materials certified by NIST. Within the Rotterdam Study [15], Licher et al. evaluated the role of Vitamin D levels as a risk factor for developing AD. Authors found that subjects with vitamin D <25 nmol/L (defined as the deficiency) had an increased risk of developing dementia, compared to those with ≥50 nmol/L (sufficiency), but this finding did not achieve statistical significance. However, the longitudinal analyses (follow-up period 13.3 years) revealed that the lower the baseline 25(OH)D levels, the higher the risk of developing AD. The Licher’s study has various plus points, consisting of robust methods: for instance, the first 5 year follow-up period was excluded from the analysis to avoid reverse causation; a sensitivity analysis excluding patients with stroke was performed; each analysis was adjusted for several confounders. Nonetheless, an electrochemiluminescence binding assay was used to measure Vitamin D, while liquid chromatography-tandem mass spectrometry (LC/MS-MS) is recommended as the gold standard assay method; also, the adoption of NIST-certified procedures and materials has been not reported.

Opposite results were obtained by Ulstein et al. [23], who reported no association between vitamin D levels and AD development. To note that the Ulstein study sample size was small (73 AD patients and 63 controls). Karakis et al. [25] analyzed 1663 non-demented subjects for a 9-years follow-up period, documenting that no association exists between 25(OH)D levels and incident AD. In this study, Vitamin D deficiency, insufficiency, and sufficiency were defined as <12 ng/mL, 12 to <20 ng/mL, and 20 to <50 ng/mL, respectively.

As it can be noted, a high heterogeneity among the cut-offs used to define Vitamin D status exists, as it has been confirmed by Balion et al. [26], who documented an association between 25(OH)D concentrations and the risk of developing AD in a meta-analysis of 35,000 subjects. However, the authors highlighted remarkable discrepancies among the studies reviewed, undermining the findings obtained.

The interpretation of the studies mentioned above should take into account some considerations. First, many drawbacks weaken the results of the studies performed, including the differences among the assay methods used to measure Vitamin D, the heterogeneity among the cut-offs used to define Vitamin D deficiency and insufficiency, the lack of internationally recognized procedures and materials, and the discrepancies among the measures used to define the cognitive function.

### 2.2. Interventional Studies

To establish a role for Vitamin D in AD, a key question is whether AD onset is preventable by increasing Vitamin D serum levels, since diagnostic biomarkers for AD are available, and predicting prognosis and treatment response is difficult due to the lack of effective therapies [34]. Randomized controlled trials (RCTs) are suitable tools to address this question, but, unfortunately, they are few and achieved debatable conclusions [35,36,37,38,39,40,41,42,43,44,45].

Generally, it could be stated that Vitamin D supplementation failed to prevent AD onset [35,36,37,38,42,43,45,46]. It is worth mentioning Rossom et al. on 4143 older women free from dementia, receiving 400 IU or placebo, reporting a similar cognitive decline incidence between the treatment and placebo groups. Authors proved that exogenous Vitamin D has no impact on dementia development risk [37]. Although Jia et al. gained opposite findings, it should be noted that the sample size of the Jia study was smaller (210 patients) and the follow-up period short (12 months vs. 7.8 years in Rossom’s study) [39]. Some authors reported that Vitamin D could improve cognitive function combined with other compounds, like memantine [40] and medium-chain triglycerides plus L-leucine-rich amino acids [41], but also these studies had limited populations and follow-up duration. Oppositely, the Cochrane Database of Systematic Reviews has recently published the exciting findings of Rutjes et al., who performed a meta-analysis to assess the impact of vitamins supplementation on cognition in healthy individuals. Authors found no evidence of a significant influence of vitamin supplementation in the risk of cognitive decline, and, importantly, revealed that many studies reporting an effect of Vitamin D in cognitive performance had a low grade of certainty, that is a marked difference between the estimated effect and the true one [45]. In 2020, Bischoff-Ferrari et al. carried out an RCT in 1900 subjects within the DO-HEALTH RTC, evaluating the impact of Vitamin D supplement on the Montreal Cognitive Assessment (MoCA) in a 3-year follow-up. Authors conclude that Vitamin D has no impact on cognitive function improvement [42]. Although other authors gained different results in the same year [44], here again, the study sample and follow-up sharply differ between the two studies, having Bischoff-Ferrari’s RCT a larger population and a longer follow-up.

When evaluating interventional studies, the impact of AD lengthy latency period should be taken into account, which further hinders univocal interpretation of the potential role for vitamin D in this disease. Indeed, during the course of AD, modifications of the mechanisms underlying the progression occur, which increases intricacy in understanding the pathophysiology and, in turn, of the candidate risk factors of the disease.

Taken together, RCTs suggest that Vitamin D supplementation does not influence cognition, regardless of the dose of the administration [46].

## 3. Conclusions

There is no uncertainty that Vitamin D takes part in normal brain function, and low Vitamin D levels can occur among demented patients. However, this finding’s clinical and laboratory significance remains unclear, also due to several drawbacks of the available studies, weakening their results and hampering concluding. Taken the evidence of past and recent literature with the appropriate cautions, Vitamin D cannot be considered a reliable biomarker of AD, since measuring the biomarker does not improve diagnosis and prognosis in these patients. Also, no clear evidence on the role of low Vitamin D levels as a risk factor for the disease exists since interventional studies on this topic are few and findings are inconsistent. Preventing the onset of AD by modifying Vitamin D levels seems too good to be true.

## Figures and Tables

**Table 1:** Characteristics of studies included in the analysis of vitamin D deficiency and the risk developing Alzheimer Disease.

Author & Publication Year	Ref.	Study Type	No. Patients (Total)	Follow-Up Duration	Vitamin D Deficiency Cut-Off	Vitamin D Assessment Method	Use of Procedure NIST	Conclusion
Afzal, 2014, Denmark	[19]	Prospective	10186	30 years	25 nmol/L	ECLIA	Not reported	Lower vitamin D concentrations increase the risk of developing AD
Aguilar-Navarro, 2019, Mexico	[21]	Cross-sectional	208	Not reported	20 ng/mL	CMIA	Not reported	Vitamin D deficiency is associated with AD
Buell, 2010, France	[16]	Cross-sectional	318	Not reported	10 ng/mL	RIA	Not reported	Vitamin D deficiency is associated with AD
Duchaine, 2020, Canada	[11]	Prospective	661	5.4 years	50 nmol/L	CLIA	Not reported	No association between 25(OH)D and AD
Feart, 2017, France	[17]	Prospective	916	12 years	25 nmol/L	CMIA	Not reported	Association between lower vitamin D concentrations and increased risk of AD
Karakis, 2016,	[25]	Prospective	1663	9 years	12 ng/mL	RIA	Not reported	No associations between vitamin D levels and incident of AD
Lee, 2020, Korea	[13]	Prospective	2990	Not reported	10 nmol/L	CMIA	Not reported	No direct correlation between VitD deficiency and cognitive impairment
Licher, 2017, Netherlands	[15]	Prospective	6220	13.3 years	25 nmol/L	ECLIA	Not reported	Lower vitamin D concentrations increase the risk of developing AD
Littlejohns, 2014, US	[14]	Prospective	1658	5.6 years	50 nmol/L	LC-MS	SRM certified by NIST	Vitamin D deficiency increases the risk of developing AD
Manzo, 2016, Italy	[12]	Cross-sectional	132	Not reported	10 ng/mL	Not reported	Not reported	No association between vitamin D deficiency and cognitive impairment
Olsson, 2017, Sweden	[24]	Prospective	1182	18 years	50 nmol/L	HPLC-MS	Not reported	No association between baseline vitamin D status and long-term risk of dementia
Shih, 2020, China	[22]	Cross-sectional	146	Not reported	20 ng/mL	RIA	Not reported	Reduced serum 25(OH)D levels are associated with lower MMSE scores in patients with mild AD

CLIA: Chemiluminescence-immunoassay; CMIA: ChemiluminescentMicroparticle immunoassay; ECLIA: electrochemiluminescent immunoassay; HPLC: High-performance liquid chromatography-mass spectrometry; LC-MS: Liquid chromatography tandem mass spectrometry; MMSE: Mini-Mental State Examination; NIST: National Institute for Standard and Technology; RIA: Radioimmunoassay; SRM:standard reference materials.
